# The Layer + Model: Incorporating Psychosocial Considerations into Hip Preservation Surgery

**DOI:** 10.1007/s12178-025-09994-3

**Published:** 2025-11-20

**Authors:** A. L. Gornitzky, I. Zaltz, M. J. Hartwell, A. Bedi, B. T. Kelly

**Affiliations:** 1 Department of Orthopaedics, C.S. Mott Children’s Hospital, University of Michigan, 1540 E. Hospital Drive, SPC 4241, Ann Arbor, MI USA; 2 Hospital for Special Surgery, New York, NY USA

**Keywords:** Layer Concept, Psychosocial considerations, Hip preservation surgery, Dysplasia, Femoroacetabular impingement, Non-mechanical pain

## Abstract

**Purpose of Review:**

The Layer Concept is a widely used model that provides an excellent anatomic framework with which to systematically diagnose and treat non-arthritic hip pain. More recently, there is a growing body of evidence highlighting the significant impact that psychosocial factors can have on both the presentation of various hip disorders and clinical outcomes following hip preservation surgery (HPS). Herein we propose the Layer + Model as a simple modification to help clinicians better diagnose and treat patients presenting with non-arthritic hip pain.

**Recent Findings:**

Building on the original four layers (osseous, inert, contractile, neuromechanical), the Layer + Model adds in a fifth layer, the psychosocial layer, to contextualize the numerous non-mechanical factors that influence perceived pain and patient-reported outcomes. Such psychosocial variables can include everything from pain level and chronicity to quality of life, mental health, social and family health, cultural contributions and many more factors that we are just learning about. This systematic review summarizes the existing evidence supporting the inclusion of a psychosocial layer. Additionally, we highlight early multidisciplinary efforts aimed at addressing each of these factors around the time of HPS.

**Summary:**

For patients presenting with non-arthritic hip pain, a complete understanding of all five layers is essential to make an accurate diagnosis and subsequently customize therapeutic recommendations to each patient’s unique needs. By recognizing the importance of such psychosocial factors, the Layer + model may also help to support the continued research and development of multidisciplinary strategies to screen for (and treat) psychosocial risk factors around the time of HPS.

At its core, hip preservation surgery is built upon the notion that the intrinsic mechanical environment of the hip is the primary driver of symptomatology. In turn, surgery aims to identify and correct the abnormal mechanics about the hip to reduce pain, treat dysfunction and slow (or prevent) the progression to osteoarthritis.

This is akin to the biomedical model of pain control, which posits that nociception is the physiology of actual or potential tissue damage, whereas pain is the cognitive and emotional response to nociception [1–3]. In other words, there is a direct causal relationship between abnormal anatomy and perceived pain. The more abnormal someone’s anatomy is, or the larger their reconstructive surgery is, the more pain they would be expected to experience. Translated to the hip, this model assumes that the underlying anatomic abnormality is the *only* contributing factor to a patient’s symptoms.

However, a growing body of evidence suggests that there are numerous non-mechanical factors in hip preservation surgery (and orthopaedics at large) that influence perceived pain and patient-reported outcomes [4–11]. Just as different people respond differently after an identical procedure, so to do people respond differently to an identical degree of osteoarthritis, instability, impingement or other anatomic variants. People are more than their anatomy. This makes sense when considering the definition of pain as the cognitive *and emotional* response to nociception – the emotional response is going to be inherently different from person to person.

In contrast, the biopsychosocial model suggests that biological, psychological and social factors together influence the pain that someone experiences [1, 12–14]. Applied to the hip, this model recognizes that the patient and their individual narratives, experiences and expectations are central to the diagnostic, therapeutic and recovery process. As Dr Millis noted in his article on the field of Hipology, “Hip surgery is often a major traumatic event for the patient and family, with a psychological, emotional and sometimes financial burden that may be greater than the obvious physical burden” [15].

Building on this, there is an emerging body of evidence on the significant impact various psychosocial factors may have on clinical outcomes following hip preservation surgery. In a prospective study looking at baseline psychological health in 58 patients undergoing hip preservation surgery, Podeszwa and colleagues found that comorbid anxiety and depression were quite common, with up to one-third of patients reporting maladaptive behaviors secondary to chronic pain and long-term functional impairment that adversely affected post-operative outcomes [5]. Looking further, they found that the only significant predictor of the amount of pain at 6 months after surgery were pre-operative pain and pre-operative mental health [16]. The more mental symptoms reported pre-operatively, the higher the predicted pain scores at six months post hip preservation surgery.

Psychosocial factors also come into play when projecting anticipated recovery following HPS. Looking at the post-operative recovery trajectories of 421 patients undergoing PAO as part of the ANCHOR cohort, Sieberg et al. found that there were no differences in pre-operative demographic, radiographic or surgical characteristics between patients with low pain and high pain [6]. Instead, the authors found that the only predictors of post-operative pain trajectories were higher levels of baseline pain and lower levels of baseline quality-of-life and functional status.

As surgeons, we are trained to evaluate a patient, generate a differential diagnosis, and propose a treatment to address that specific issue. As described by Draovitch and colleagues, the layer concept is a systemic means to determine which structures about the hip are the source of the pathology, which are the pain generators, and how you can use that information to best implement treatment [17]. Essentially, it’s a conceptual framework for all the inter-related mechanical and biological systems that clinicians can utilize in everyday practice to identify the most likely etiology of a patient’s symptoms. There are four layers (osseous, inert, contractile & neuromechanical), and each is made up of various anatomic structures which together serve a common purpose. For example, the inert layer includes the capsule, labrum, ligamentous teres and ligamentous complexes which together provide static stability, while the contractile layer includes the musculature crossing the hip, the lumbosacral muscles and the pelvic floor which work together to provide dynamic stability. At its core, the Layer Model assumes that structure alone dictates treatment. Just like the biomedical model of pain, it assumes a direct relationship between abnormal anatomy and pain without fully accounting for the significant mediating impact psychosocial variables can have on clinical outcome and symptomology.

Building on this foundational work, we propose the Layer + model (Fig. 1), which recognizes and accounts for the significant contribution of psychosocial factors to clinical outcomes following hip preservation surgery. Such psychosocial variables can include everything from pain level and chronicity to quality of life, mental health, social and family health, cultural contributions and many more factors that we are just learning about. Although not biomechanical in nature, each of these factors can still have a profound effect on clinical outcomes following hip preservation surgery.

**Fig. 1:**
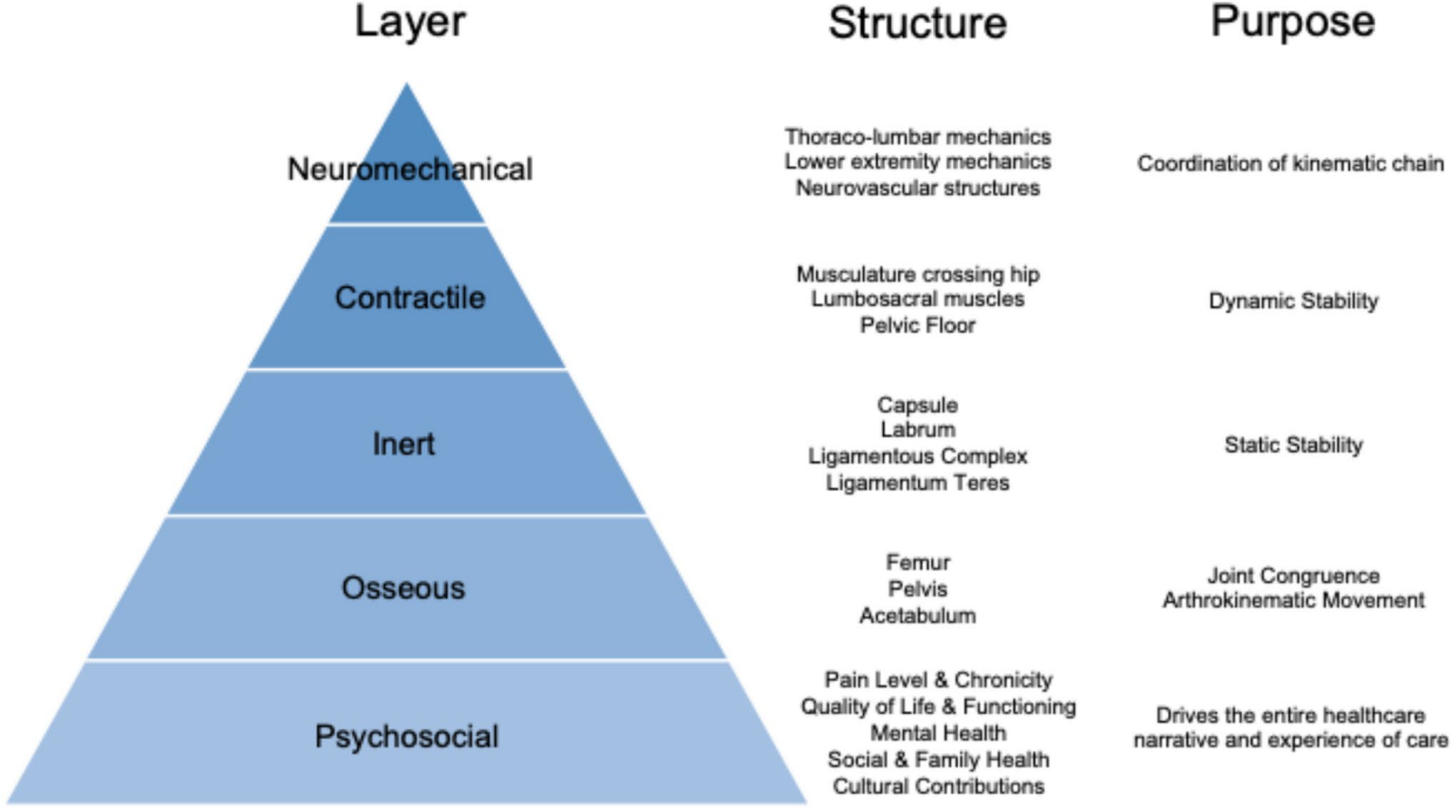
Layer + Model. This model includes a psychosocial layer which recognizes and accounts for the significant contribution of psychosocial factors to clinical outcomes following hip preservation surgery

Extending beyond anatomic factors, these psychosocial considerations also have the potential to drive the entire healthcare narrative and broader experience of care. In many ways, they should be conceptualized as the all-important base layer of the pyramid that supports and influences the entire recovery trajectory (Fig. 2). It is crucial to remember that hip preservation surgery does not actually address these psychosocial variables – it only corrects the mechanical forces about the hip joint. As a result, if there are potential psychosocial considerations driving the pain and functional impairments that a patient is experiencing, even if only partially, it may be worth addressing these variables prior to surgical treatment of a mechanical hip disorder.

**Fig. 2:**
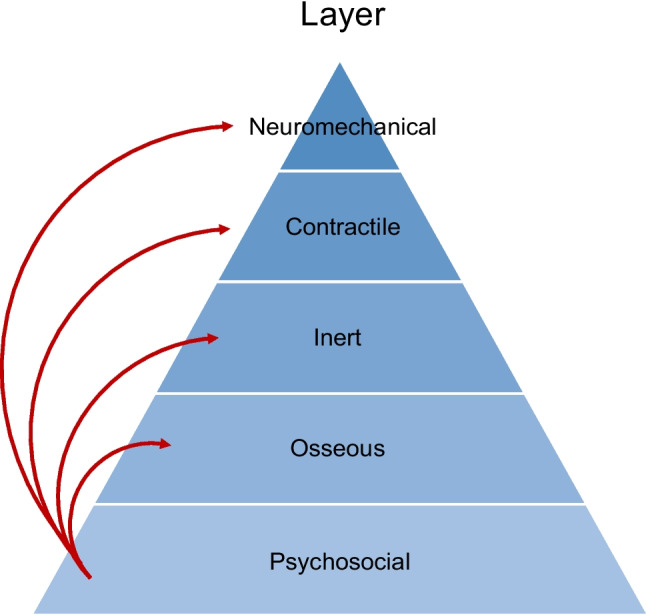
Impact of the Psychosocial Layer. Visual conceptualization of the psychosocial layer as the all-important base layer of the pyramid that supports and influences the entire recovery patient experience with non-arthritic hip pain

Just like the original Layer model, the Layer + modification is not a classification scheme. Rather, each structure, or layer, has a distinct and independent contribution to overall hip function, and influences specific treatment recommendations for specific anatomic disorders. Putting this into practice, every patient has a narrative that deserves close attention. When diagnosing and treating hip pain, “non-physical expertise, including true shared decision-making, is of equal importance” as “anatomic knowledge and technical skill” [15].

It is important to acknowledge that surgeons are not trained to manage the psychosocial aspects of patient care. [18, 19] As expert technicians, it is easier to offer surgery than to contemplate the complicated and windy pathway that is psychosocial treatment and complex care coordination. The optimal solution likely lies in building interdisciplinary teams of experts that, together, can address complex psychosocial issues. [14, 20–22] For example, Richard and colleagues reported their experiences building a collaborative and proactive care model for HPS that includes standardized pre-operative psychological intervention, physical therapy and nursing education to outline expectations and goals and provide additional psychological services [23]. Such multidisciplinary efforts may have promising results, as adolescents in their cohort all had improved psychological function, resiliency and mental health, which in turn lead to improved optimism and self-efficacy that positively correlated with increased mobility and return to activity. More work is needed to identify effective strategies that clinicians can use when they don’t have access to such resources.

In conclusion, chronic pain, poor mental health and decreased quality-of-life are all associated with poor outcomes following HPS. Building upon the previously described Layer model [17], the Layer + model better accounts for psychosocial considerations when diagnosing and treating hip pain. By highlighting the importance of such psychosocial factors and placing them on an equal footing with the many biomechanical factors known to impact hip health, the Layer + model may also help to support the continued research and development of multidisciplinary strategies to screen for (and treat) psychosocial risk factors around the time of HPS.

## Key References

- Podeszwa DA, Richard HM, Nguyen DC, De La Rocha A, Shapiro EL. Preoperative psychological findings in adolescents undergoing hip preservation surgery. J Pediatr Orthop. 2015;35:253–7.
  - This prospective cohort study found that comorbid anxiety and depression were quite common in HPS patients, with up to one-third of patients reporting maladaptive behaviors secondary to chronic pain and long-term functional impairment that adversely affected post-operative outcomes.
- Sieberg CB, Klajn J, Wong C, Bowen G, Simons LE, Millis MB. Predictors and trajectories of chronic postoperative pain following hip preservation surgery. J Hip Preserv Surg. 2017;4:45–53.
  - This large cohort study of 421 patients undergoing PAO as part of the ANCHOR cohort described three common post-operative pain trajectories, and found that the only significant predictor of the amount of pain at 6 months after surgery were pre-operative pain and pre-operative mental health.
- Wagener N, Löchel J, Hipfl C, Perka C, Hardt S, Leopold VJ. Psychological status affects postoperative quality of life, function, and pain after periacetabular osteotomy. Bone Jt Open. 2023;4:758–65.
  - This study of 101 patients undergoing PAO demonstrated that psychological distress, depression and somatization disorders each affected health-related quality-of-life, perceived joint function and sports ability following surgery. However, satisfaction with surgery was not affected.
- Rabbitts JA, Palermo TM, Zhou C, Meyyappan A, Chen L. Psychosocial Predictors of Acute and Chronic Pain in Adolescents Undergoing Major Musculoskeletal Surgery. J Pain. 2020;21:1236–46.
  - This prospective study of 119 adolescents undergoing major musculoskeletal surgery found that presurgery pain severity predicts acute postsurgical pain, while depressive symptoms and poor sleep quality predict chronic postsurgical pain. Approximately 20% of youth met criteria for chronic pain at 4 months post-operatively.
- Millis MB. Hipology 2023: Science, Philosophy, and Craft. HSS J. 2023;19:467–72.
  - This narrative review from a long-time international thought-leader in the HPS space highlights the importance of the biopsychosocial model to clinical hip dysfunction, and offers sage advice on the importance of incorporating a holistic, multidisciplinary approach into the treatment of patients with non-arthritic hip pain.
- Richard HM, Cerza SP, De La Rocha A, Podeszwa DA. Preoperative mental health status is a significant predictor of postoperative outcomes in adolescents treated with hip preservation surgery. J Child Orthop. 2020;14:259–65.
  - This prospective study of 58 HPS candidates showed that pre-operative pain and mental health symptoms are both predictive of post-operative pain. Highlighting the significant potential of coordinated psychological treatment, they also showed that HPS candidates evaluated preoperatively by psychology, as part of an integrated treatment approach, demonstrated statistically significant improvements in pain, health-related quality of life and mental health symptoms.
- Draovitch P, Edelstein J, Kelly BT. The layer concept: utilization in determining the pain generators, pathology and how structure determines treatment. Curr Rev Musculoskelet Med. 2012;5:1–8.
  - This foundational paper introduced and described the Layer concept.

## Data Availability

No datasets were generated or analysed during the current study.
